# Recyclability Analysis of Starch Thermoplastic/Almond Shell Biocomposite

**DOI:** 10.3390/polym13071159

**Published:** 2021-04-05

**Authors:** Ana Ibáñez-García, Asunción Martínez-García, Santiago Ferrándiz-Bou

**Affiliations:** 1 AIJU, Technological Institute for Children’s Products & Leisure, 03440 Ibi, Alicante, Spain; sunymartinez@aiju.es; 2 Technological Institute of Materials (ITM), Universitat Politècnica de València (UPV), Plaza Ferrándiz y Carbonell 1, 03801 Alcoy, Alicante, Spain; sferrand@mcm.upv.es

**Keywords:** almond shell, starch thermoplastic polymer, biodegradable, biocomposite, recycling, reprocessing cycle, injection molding, natural filler

## Abstract

This article is focused on studying the effect of the reprocessing cycles on the mechanical, thermal, and aesthetic properties of a biocomposite. This process is based on starch thermoplastic polymer (TPS) filled with 20 wt% almond shell powder (ASP) and epoxidized linseed oil (ELO) as a compatibilizing additive. To do so, the biocomposite was prepared in a twin-screw extruder, molded by injection, and characterized in terms of its mechanical, thermal, and visual properties (according to CieLab) and the melt flow index (MFI). The analyses carried out were tensile, flexural, Charpy impact tests, differential scanning calorimetry (DSC), thermogravimetric analysis (TGA). The effects of the reprocessing were also studied for the biodegradable unfilled TPS polymer. The results showed that TPS and TPS/ASP biocomposite suffer changes progressively on the properties studied after each reprocessing cycle. Furthermore, it was observed that the addition of ASP intensified these effects regarding TPS. However, in spite of the progressive degradation in both cases, it is technically feasible to reprocess the material at least three times without needing to incorporate virgin material.

## 1. Introduction

Recently, there has been a considerable interest in the development of biobased polymers to decrease dependency on petroleum-based polymers due to environmental concerns. Biopolymers derived from renewable resources have a wide range of applications in different industries due to their specific characteristics. As a result of researches and advances in R&D, the properties of these materials have been improved, and this ushered in new markets ranging from packaging, food service, consumer electronics, automotive, agriculture/horticulture, and toys to textiles. Although packaging, either rigid or flexible, remains the largest field of application for these materials, with almost 53 percent (1.14 million tons) of the total bioplastics market in 2019 [1]. Despite this interest, there are serious doubts about the behavior of these plastics during processing. One of them is the possibility of reprocessing the material generated by defective production or cut parts (sprues, distribution channels, etc.).

Traditionally, the reprocessing of the thermoplastic materials led to a significant deterioration of the material properties. This deterioration was the result of the decrease in molecular weight caused by the breakage of the polymeric chains that occurs when the material is subjected to a high shear process. On the other hand, loss of mechanical and thermal properties as well as discolorations are some of the most common degradation problems in polymers as a result of the recycling process. Recycled polymers must possess a set of minimum performance characteristics to meet specific requirements after the recycling process. Therefore, it is of great interest to know the impact of successive reprocessing cycles and conditions on the physical, mechanical, and thermal properties of polymers.

Biodegradable materials, such as polylactic acid (PLA), polyhydroxyalkanoate (PHA), starch-based polymer (TPS), are thermoplastic materials and, therefore, they can be reprocessed. However, these materials are much more sensitive to thermo-mechanical degradation [2].

Mechanical recycling has been studied in biodegradable polyesters, such as PLA, for several reasons. On the one hand, because plastic waste is generated during the transformation process [3]. On the other hand, to extend the useful life of the material, the possibility of reusing post-consumer waste has been studied [4].

The reprocessing of PLA in different transformation processes has also been studied. One of the processes evaluated was the continuous extrusion up to ten times. The tensile properties of PLA do not depend significantly on the number of extrusion cycles, while the impact strength and viscosity clearly decrease by increasing extrusion cycles [4]. On the contrary, when the material is processed by injection molding, the values of tensile strength, breaking strain, hardness, and viscosity decreased progressively from the first processing [3]. This loss of mechanical properties is due to the polymer chain breaking during processing, which results in a significant decrease in molecular weight [2]. Another study that additionally corroborates the loss of mechanical properties with the number of injection cycles showed changes in the color of injected parts, which intensifies as the processing cycles increase [5]. Furthermore, the effect of drying a PLA/PBAT (polybutylene adipate terephthalate) blend before reprocessing has also been studied recently. Rheological characterization showed that when the sample was processed after drying, less degradation was observed, as hydrolysis degradation of the components was minimized. However, regardless of whether the mixture was dried or not, after the fifth extrusion process, the mechanical properties did not decrease significantly [6].

Unlike PLA, few studies are available on the mechanical recycling of other pure biopolymers, such as PHA or TPS. PHA can be recycled mechanically, assuming the loss of molecular weight and mechanical properties [2]. As for TPS, it was found through a comparative study that the reprocessing capacity depends on the chemical structure of the polymer. While one of the references, Mater-Bi TF01U/095R, could be reprocessed up to ten times, the other one, Mater-Bi YI014U/C, was not viable due to the large loss of mechanical properties of the material from the first reprocessing cycle [7].

When it comes to the mechanical recycling of biocomposites, they have also been studied. In general, it is expected that biopolymers reinforced with natural fibers are less likely to be mechanically recycled than the material itself, as biocomposites are more sensitive to thermo-mechanical degradation [2].

TPS is one of the most promising polymers to replace petroleum-based ones. TPS is based on different starches (wheat, corn, yucca, potato, etc.) [8]. Chemically, starch is constituted of two polysaccharides: amylose and amylopectin. It is partially crystalline and biodegradable in different media: water, soil, and compost. Depending on their ratio, mechanical properties can vary to a great extent. TPS is easy to process but very sensitive to thermal and mechanical degradation in the process and moisture. The density is higher than the most conventional thermoplastic polymers, between 1.2 and 1.5 g/cm^3^ [9]. The mechanical properties of TPS are generally inferior to those of petrochemical plastics, so it is common to find commercial grades blended with other degradable polymers (PCL (polycaprolactone), PVOH (Polyvinyl alcohol), PLA, PHA) to improve their mechanical properties without affecting their biodegradability.

Our previous work focused on TPS/ASP biocomposites, with a constant ASP content of 20 wt%, and several epoxidized vegetable oils (EVOs) were added at 5, 10, and 20 parts per hundred resin (phr) of TPS/ASP biocomposites, as additives. Said oils were epoxidized soybean oil (ESBO), epoxidized linseed oil (ELO), and epoxidized corn oil (ECO). The purpose of the work was to study the effect on the properties of injected biodegradable parts. The most optimal performance was attained for biocomposites with ELO or ESBO between 10 and 20 phr.

Taking as a reference one of the above-mentioned optimal formulations, the main objective of this present study is to study the influence of reprocessing cycles on the properties of TPS and TPS/ASP biocomposite in terms of their mechanical and thermal properties and changes in morphology, visual appearance, and melt flow index.

This study shows the degradation of TPS and TPS/ASP and establishes possible strategies for the reuse of the discarded parts generated during the manufacturing process with these materials.

## 2. Materials and Methods

### 2.1. Materials

For this study, a commercially available starch-based polymer, Mater-Bi^®^ EI51N0 of Novamont, was studied. This bio-based and biodegradable polymer has a melt flow index (MFI) of 19 g/10 min (190 °C/2.16 kg) and a density of 1240 kg/m^3^ (data provided by Novamont). This grade was selected for its properties, which are quite similar to polypropylene (PP), of high use in injection molding. Table 1 shows some properties of the as-received material.

**Figure 1:**
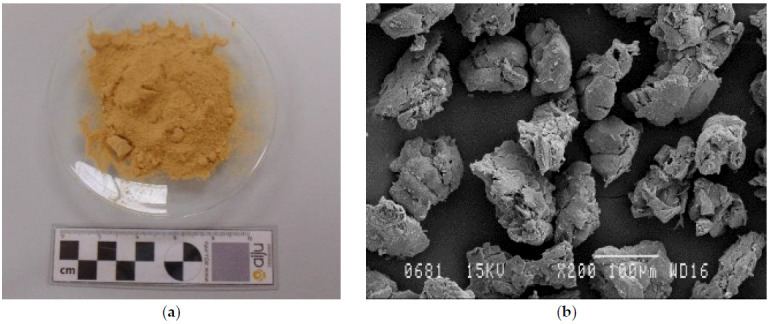
(**a**) Almond shell powder (ASP) powder, (**b**) Scanning electron microscope (SEM) image of ASP (×200 magnification and a scale marker of 100 μm).

Almond shell powder (ASP) with particle size between 0.05 and 0.125 mm provided by Hermen Europe, S.L. (Spain) was used for this study (Figure 1a). It consisted of a mixture of different almond shell varieties. Figure 1b shows that the predominant particle size range was about 0.08–0.125 mm. Table 2 shows the content of fixed carbon, volatile matter, humidity, ash, and the chemical composition of the main components of the used almond shell: hemicellulose, lignin, and cellulose, determined by thermogravimetric analysis (TGA) in previous work [10].

The compatibilization of starch polymer and ASP was carried out with epoxidized linseed oil (ELO), supplied by Traquisa S.L. (Spain). Some of its properties are shown in Table 3.

### 2.2. Experimental Procedure

#### 2.2.1. Preparation of Composites

Before processing, TPS and ASP were dried separately for 4 h at 60 °C and 24 h at 105 °C, respectively, in a SLW 115STD INOX air-circulating oven from POL-EKO (Wodzisław Śląski, Poland) to minimize its moisture and to avoid hydrolytic reactions [11].

A composite of starch-based polymer with ASP was developed using a BERSTORFF ZE26 × 44D-BASIC co-rotating twin-screw extruder (26:44 L/D). Before feeding the material into the extruder, a manual pre-mixing of the different components, Mater-Bi^®^ EI51N0/ASP/ELO, was carried out and fed into the extruder through the main hopper. The ratio of TPS/ASP was set at 20 wt% since this content has shown balanced mechanical properties and appealing aesthetics in a previous study dealing with TPS biocomposites [12]. ELO was added at 10 parts per hundred resin (phr) of biocomposite. The temperature profile was set as follows: 130-185-185-185-185 °C (from feeding zone to die). The rotating speed was 80 rpm. The extruded material was finally pelletized using an air knife.

#### 2.2.2. Injection Molding

Testing samples were molded using an injection molding machine Ergotech 110–430h/310V from DEMAG (Demag, Germany). The injection conditions used to develop recycled TPS and biocomposite TPS/AASP test specimens are listed in Table 4. After each injection cycle, the material was milled and dried at 60 °C for 6 h. Then, TPS was processed a total of four cycles and TPS/ASP biocomposite six cycles. The difference in the number of processing cycles between TPS and TPS/ASP was due to the poor quality of the specimens after TPS-4. TPS-5 presented injection defects, such as shrinkage. The nomenclature of recycled TPS and TPS/ASP biocomposite is given in Table 5. Specimens were conditioned at a temperature of 23 °C and relative humidity of 50% for at least 16 h before testing.

#### 2.2.3. Color Measurements

The influence of the reprocessing cycles on the color of the TPS and TPS/SP biocomposite specimens was recorded with a CR-200 Chroma Meter from KONICA MINOLTA (Tokyo, Japan). Moreover, the color indexes (L*, a* and b*) were measured according to the following criteria: L* = 0, darkness; L* = 100, lightness; + a* = red, − a* = Green and + b* = yellow, − b* = blue. From these coordinates, it was possible to determine the color difference associated with this space. The color variation, $∆E_{ab}^{*}$, was obtained by the following Equation (1) and compared with the color coordinates of the neat TPS (TPS-1) and TPS/ASP (TPS/ASP-1).

(1) $$∆E_{ab}^{*}=\sqrt{{\left(∆L^{*}\right)}^{2}+{\left(∆a^{*}\right)}^{2}+{\left(∆b^{*}\right)}^{2}}$$

The color change was assessed according to experimentally verified data [13]: Unnoticeable ($∆E_{ab}^{*}<1)$, only an experienced observer can notice the difference $\left(1<∆E_{ab}^{*}<2\right)$, an unexperienced observer notices the difference $\left(2<∆E_{ab}^{*}<3.5\right)$, there is a clear noticeable difference $\left(3.5<∆E_{ab}^{*}<5\right)$, and the observer notices two different colours $\left(∆E_{ab}^{*}>5\right)$.

#### 2.2.4. Mechanical Properties

The mechanical properties of TPS and TPS/ASP biocomposites were determined to study the capacity of their reprocessability.

Tensile and flexural tests were performed using an 6025 universal testing machine from INSTRON (Canton, Massachusetts, USA) with 5 kN power sensors. The tensile test was performed according to ISO 527 standard, using a crosshead speed of 1 mm/min for Young’s Modulus determination and 5 mm/min for tensile and elongation at break determination. The extensometer used was MTS 634.11F-54. Recorded values included ultimate tensile strength (UTS), Young’s modulus, and strain at break. A total of 5 specimens from each material were tested using standardized samples 1A (dogbone).

Impact tests were performed with a Resil 5.5 impact testing device (CEAST RESILIMPACTOR) with a 1 Joule hammer. Then, test samples were cut and tested according to ISO 179 standard. A total of 10 sample tests from each material was tested.

#### 2.2.5. Scanning Electron Microscopy (SEM)

The impact fracture surface obtained after a Charpy impact test was analyzed using a Jeol JSM-840 SEM system. Samples were gold-coated before analysis, and the energy of the electron beam was 20 kV.

#### 2.2.6. Thermal Analysis

The main thermal degradation parameters of biocomposites, initial degradation temperature (T_onset_), and maximum mass loss rate temperature (Tmax) were studied by TGA using a TA Instrument Q500 thermogravimetric analyzer from TA INSTRUMENTS (Delaware (New Castle, DE, USA)). Then, those samples with an average weight between 8 and 10 mg were placed in standard platinum crucibles of 70 µL. In this case, biocomposites were subjected to the following temperature program: from 30 to 600 °C under nitrogen (N_2_) atmosphere at a rate of 10 °C/min, and from 600 to 1000 °C under oxygen (O_2_) atmosphere at a rate of 10 °C/min with a purge gas flow of 10 mL/min.

Later on, thermal transitions of developed biocomposites were studied by differential scanning calorimetry (DSC) in a DSC Q200 calorimeter from TA INSTRUMENTS (New Castle, DE, USA). Then, those samples with an average weight of 8–10 mg were placed in standard aluminum crucibles and subjected to a three-step program that consisted of an initial heating cycle from 0 °C to 250 °C at a rate of 10 °C/min to remove thermal history, followed by cooling to −20 °C at a rate of 5 °C/min, and a second heating cycle to 350 °C at a rate of 10 °C/min. All the tests were run in an N_2_ atmosphere with a constant flow of 50 mL/min. Finally, thermal transitions were determined: temperature (T_c_) and enthalpy (∆H_c_) of crystallization after the cooling cycle and temperature (T_f_) and enthalpy of fusion (∆H_f_) after the second heating.

#### 2.2.7. Melt Flow Index

The determination of the melt flow index (MFI) was carried out according to ISO 1133 standard with a MFI TWELVINDEX equipment from ASTFAAR (Milan, Italy) at 190 °C and 2.16 kg. The cut time between two consecutive measurements was 15 s.

## 3. Results

### 3.1. Visual Aspect

**Figure 2:**
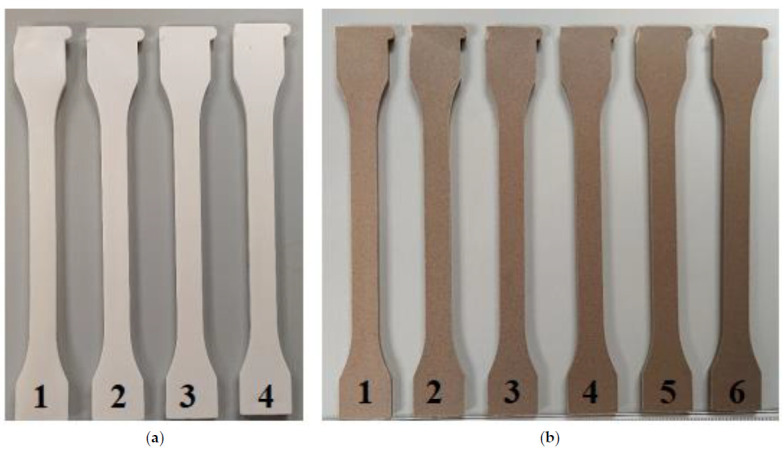
Visual aspect of the injected specimens subjected to different reprocessing cycles. (**a**) Starch thermoplastic polymer (TPS) (**b**) TPS/ASP biocomposite.

The appearance of the injected specimens of TPS and TPS/ASP after the different reprocessing cycles are gathered in Figure 2a,b, respectively. Table 6 summarizes the color indexes (L*, a* and b*), and the color variation measured by $∆E_{ab}^{*}$, with respect to TPS and TPS/ASP biocomposite with only the first processing cycle (TPS-1 and TPS/ASP-1, respectively).

The reprocessing cycles had more effect on the change in color of TPS/ASP biocomposite than in the unfilled TPS. As expected, the $L^{*}$ value decreased (white to black) in both cases. a* and b* coordinates changed progressively after each reprocessing cycle. Moreover, after the second processing cycle, the obtained $∆E_{ab}^{*}$ value of TPS was 1.00 (TPS-2). After the third reprocessing cycle, the value slightly increased to 1.91 (TPS-3); only an experienced observer could notice the difference in color [13]. However, in the case of TPS/ASP, after the second processing cycle, the value obtained of $∆E_{ab}^{*}$ was 3.96 (TPS/ASP-2), which resulted in samples whose change in color could be noticed by an observer (3.5 < $∆E_{ab}^{*}\;$< 5). The color variation clearly showed an increasing tendency in reprocessing, as expected due to degradation [13]. The $∆E_{ab}^{*}$ was more remarkable and noticeable for TPS/ASP biocomposite.

### 3.2. Thermal Properties

**Figure 3:**
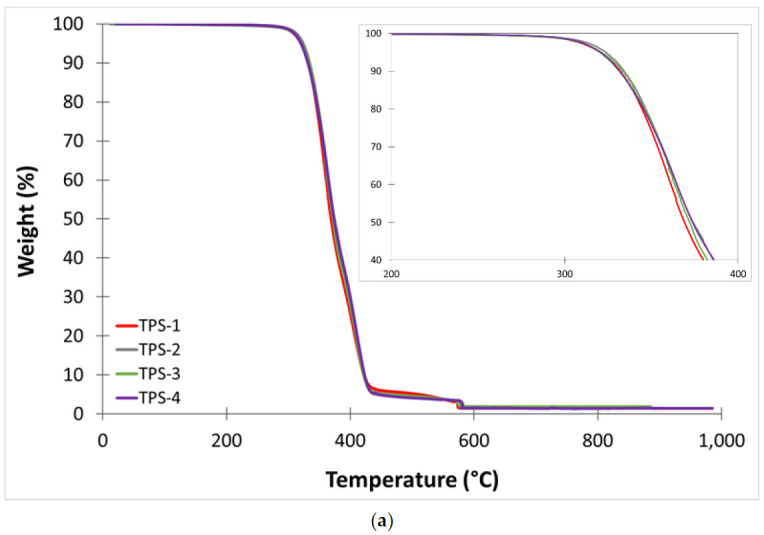

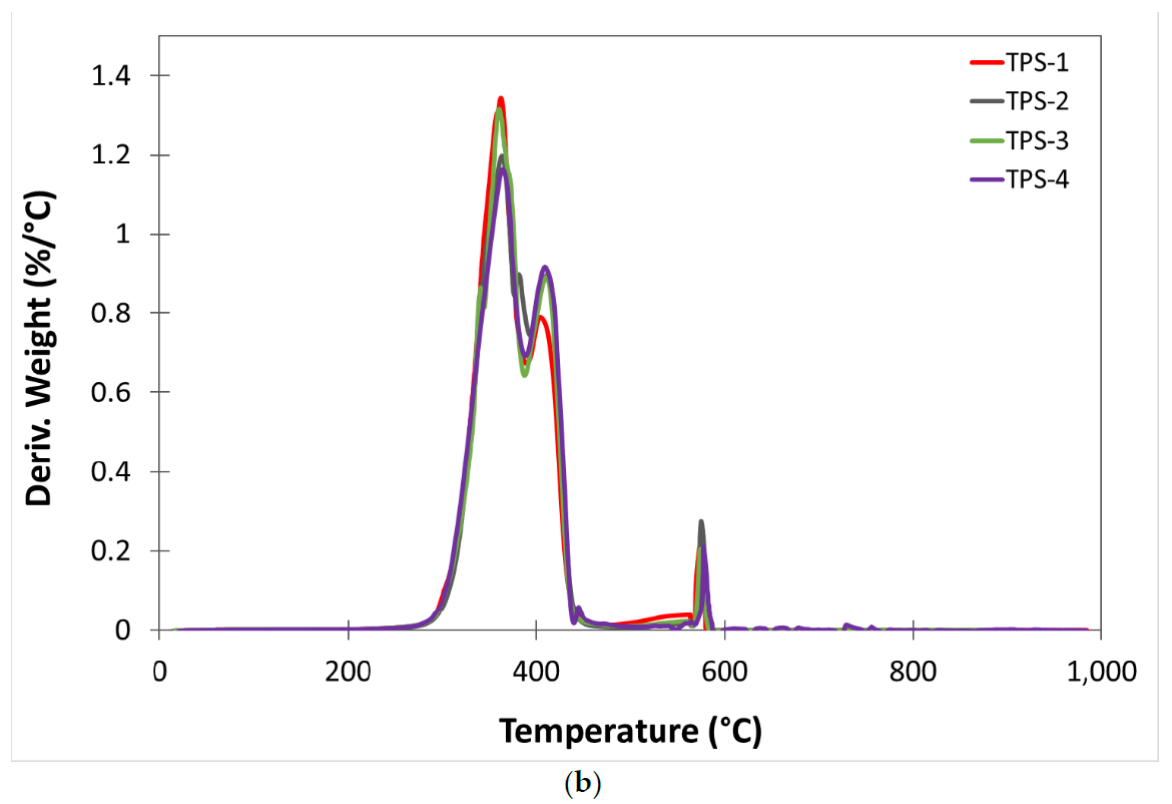
(**a**) Thermogravimetric analysis (TGA) thermograms corresponding to different reprocessing cycles of TPS (**b**) first derivative (DTG) curves.

**Figure 4:**
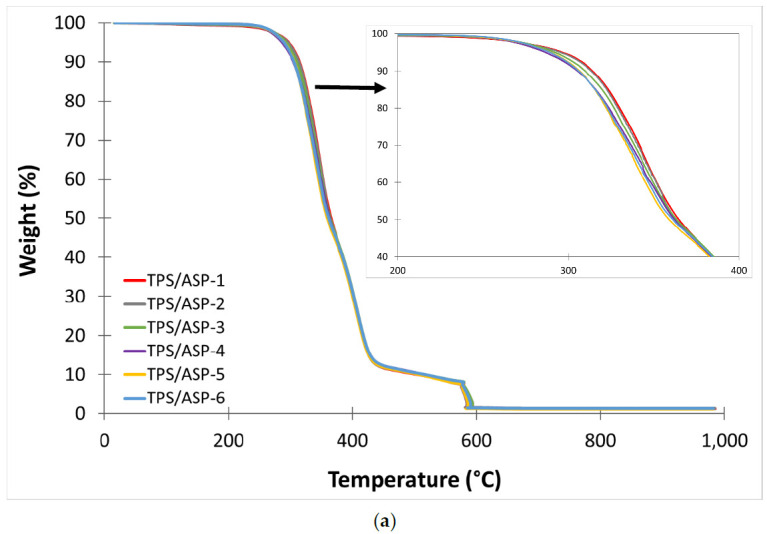

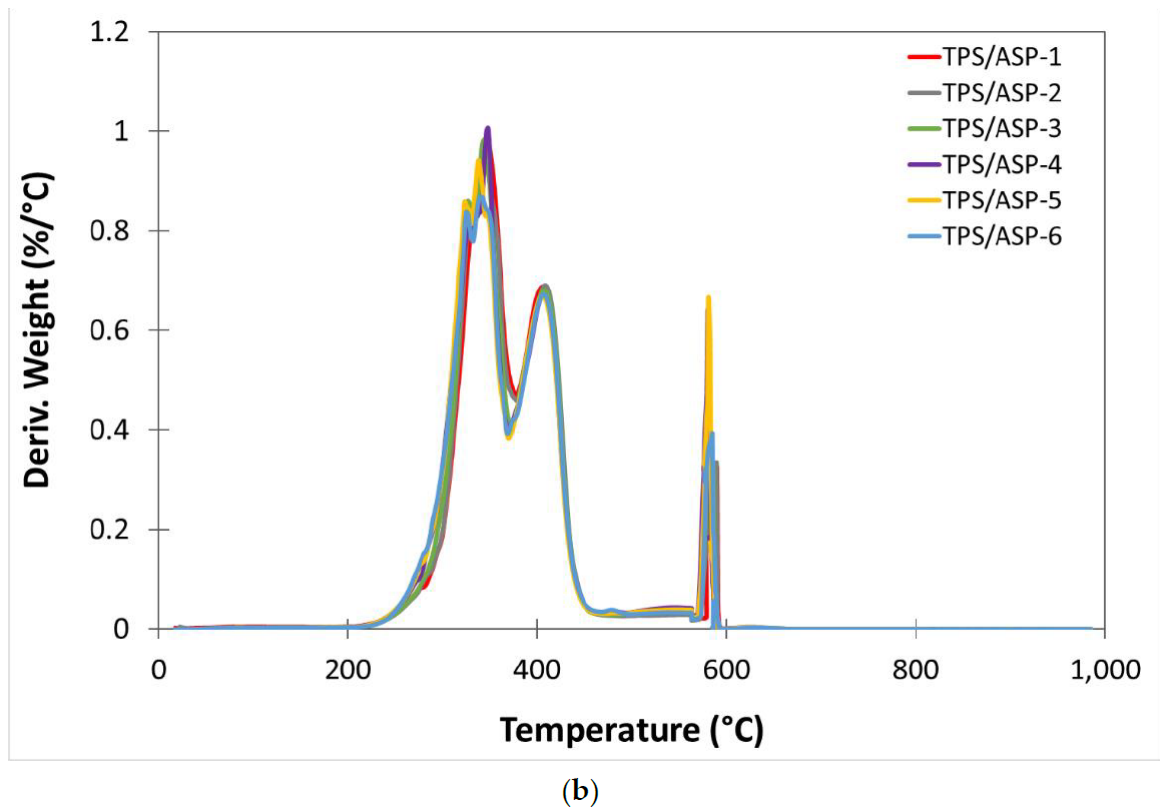
(**a**) TGA thermograms corresponding to different reprocessing cycles of TPS/ASP biocomposite (**b**) first derivative (DTG) curves.

Table 7 shows the main thermal degradation parameters, initial degradation temperature (T_onset_), and maximum mass loss rate temperature (T_max1_ and T_max2_) obtained by TGA. Figure 3 and Figure 4 show the TGA curves of TPS and TPS/ASP biocomposite, respectively, obtained after different reprocessing cycles. Furthermore, all samples presented a two-step process because the thermogravimetric analysis was carried out under an N_2_ and O_2_ atmosphere. When it comes to the comparative TGA curves of TPS samples obtained after different reprocessing cycles, those suggested no significant changes in the thermal degradation parameters since the curves overlapped. TPS-1 presented moderate thermal stability with T_onset_, and T_max1_ of 326.1 °C and 362.1, respectively. It is noticeable that, after the fourth injection cycle, the values remained almost invariable. This indicated that the reprocessing cycles did not have a significant effect on thermal degradation at the current processing temperatures. Regarding the TGA thermograms of TPS/ASP, the addition of ASP reduced the thermal stability of the biocomposite because almond shells degrade faster than polymer matrix [10,12], and T_onset_ and T_max1_ moved towards a lower temperature. T_max2_ remained practically unchanged. However, in a previous study, which is in the process of publication, it was found that the addition of 10 phr ELO had a positive effect on the thermal stability of this type of biocomposite, increasing T_onset_ and T_max1_ by 6 °C: TPS/ASP-1 biocomposite presented a T_onset_ of 315.0 °C and T_max1_ of 346.0 °C. After the second injection cycle, T_onset_ decreased by 9 °C and continued to decrease progressively after each reprocessing cycle. Nevertheless, the T_max1_ remained almost invariable until the fifth injection cycle (340.6 °C).

**Figure 5:**
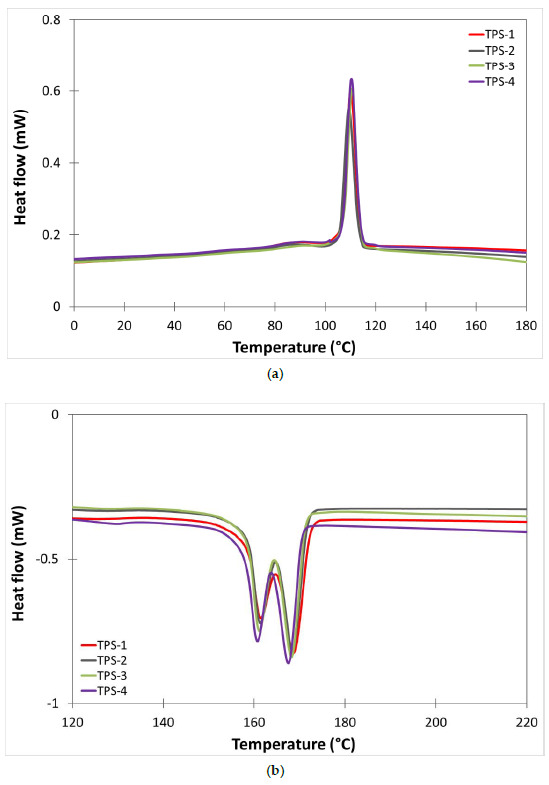
Comparative plot of differential scanning calorimetry (DSC) curves of TPS after different reprocessing cycles: (**a**) cooling cycle, (**b**) second heating cycle.

**Figure 6:**
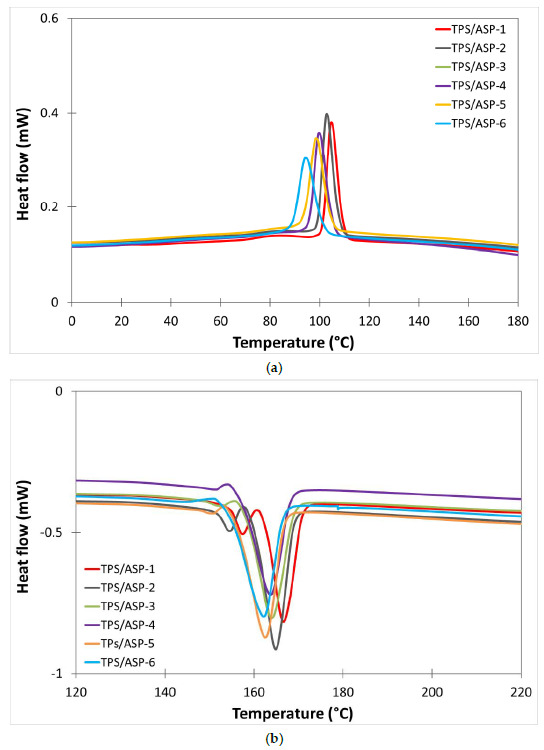
Comparative plot of DSC curves of TPS/ASP after different reprocessing cycles: (**a**) cooling cycle, (**b**) second heating cycle.

To obtain the main thermal transition of the material a differential scanning calorimetry was used. Figure 5 and Figure 6 show the DSC curves achieved from the cooling and second heating scans of TPS and TPS/ASP biocomposite samples, respectively, obtained after different reprocessing cycles. In addition, Table 8 summarizes the most relevant thermal parameters obtained from the cooling and second heating of the samples subjected to different reprocessing cycles. Bastioli et al. [14] described Novamont’s starch-based technology as a process that could destroy amylose and amylopectin from starch. Thus, the main endothermic (T_m1_ y T_m2_) and exothermic (T_c_) peaks were related to the melting and the cold crystallization of the crystalline structure of the synthetic biodegradable polymer presented in Mater-Bi, respectively. After the first injection run (TPS-1), TPS presented a bimodal endothermic peak (Figure 5b). The first one, smaller, at around 161.4 °C (T_m1_), and the second one, more pronounced, at 168.5 °C (T_m2_). These are associated with the fusion of the crystalline structure as a result of chain scission caused by different resistance to heat. The TPS-1 thermogram corresponding to the cooling scan presented a unique main exothermic peak around 109.5 °C (Figure 5a). As can be observed, after different reprocessing cycles, the thermal transitions of TPS, such as T_c_, T_m_, and their respective enthalpy, did not present significant changes. The addition of 20 wt% produced a slight reduction in all parameters. After the first injection run, TPS/ASP-1 also presented a bimodal endothermic peak. The melting point temperature, T_m1_, decreased from 161.4 °C to 157.1 °C and the T_m2_ from 168.5 °C to 166.4 °C. Regarding the normalized melting enthalpy (ΔH_m_), it decreased from 23.8 to 17.2 J/g. These results indicated that the addition of almond shell to the starch-based polymer decreases the crystallization of the molecular chains [12]. After the second injection cycle (TPS/ASP-2), T_m1_ and T_m2_ decreased 2–3 °C compared to TPS/ASP-1 and continued to decrease progressively after each reprocessing cycle until 144.6 °C and 162.7 °C (Figure 6b), respectively. The decrease in the values may be attributed to the higher mobility of the polymer chains as a result of the reduction in the molecular weight during the recycling process. In addition, it was noticed that the crystallization temperature (T_c_) slightly decreased upon adding ASP, thus indicating that the presence of ASP made crystallization start later, compared to as-received TPS. Furthermore, it was noticed that the crystallization temperature and enthalpy of TPS/ASP decreased progressively after each reprocessing cycle (Figure 6b). This indicated a loss of crystalline structure and a major difficulty in the crystallization process (i.e., starting at a lower temperature) as the number of processing cycles was increased [15].

### 3.3. Mechanical Properties

Table 9 shows a summary of the tensile properties of both TPS and TPS/ASP biocomposite after each reprocessing cycle. The TPS-1 presented Young’s modulus of 1658 MPa and tensile strength of 39.4 MPa. As can be seen in Table 9, the number of reprocessing cycles influenced the tensile properties, decreasing the values progressively. In particular, after the third processing cycle, Young’s modulus significantly decreased to 1184 MPa. Regarding the tensile and elongation at break, changes were more noticeable after the fourth processing. That indicated that the material was less ductile.

In a previous study, the addition of 20 wt% of ASP produced an increment in Young’s Modulus and a reduction in the rest of the tensile parameters (tensile and elongation at maximum strength, tensile, and elongation at break) [12]. However, in this work, as can be observed in Table 8, Young’s modulus of TPS/ASP biocomposite was lower than the unfilled TPS. The addition of ELO increased the flexibility of the biocomposite, reaching values of Young’s modulus even lower than those of the as-received TPS. Thus, the tensile and elongation of the TPS/ASP biocomposite decreased due to its progressive lack of capacity to sustain deformation. When it comes to the effect of reprocessing TPS/ASP biocomposite in tensile properties, it did not have a significant effect on Young’s modulus, which indicated similar rigidity. The rest of the parameters (tensile and elongation strength/break) kept similar values to TPS/ASP-1 until the fourth reprocessing.

**Figure 7:**
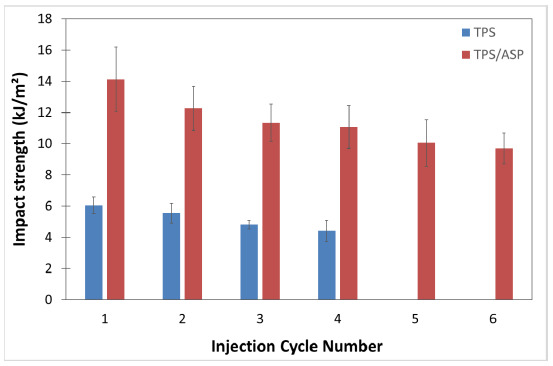
Charpy Impact strength corresponding to injection-molded samples of TPS and TPS/ASP biocomposite subjected to different reprocessing cycles.

Figure 7 shows the evolution of impact strength in both the reprocessed TPS and TPS/ASP biocomposite. The impact tests of TPS were carried out with notched samples because the material presented a high strength and did not break, even after the applied reprocessing cycles. The as-received TPS (TPS-1) presented an impact strength of 6.0 kJ/m^2^. This value barely decreased after the second injection cycle (TPS-2). After the third and fourth injection cycle, a slight reduction in energy absorption capacity was attained, reaching values around 4.5 kJ/m^2^. The reprocessing cycles did not have a significant effect on the impact strength. This behavior was observed in other grades of starch-based polymer (Solanyl, [16]). The addition of 20 wt% of ASP drastically affected the impact strength. While the TPS samples needed notching to do the test, the TPS/ASP biocomposite did not. The TPS/ASP biocomposite showed an impact strength of 14.13 kJ/m^2^. After the second reprocessing cycle, the impact strength decreased until 12.26 kJ/m^2^. Additionally, an evident reduction in energy absorption capacity was attained when increasing the reprocessing cycles, reaching values down to 9.70 kJ/m^2^ (TPS/ASP-6). This behavior was observed in PLA systems with cellulose fibers [17,18,19]. Finally, this loss in the impact strength can be attributed to the polymer degradation occurring during each thermo-mechanical recycle.

**Figure 8:**
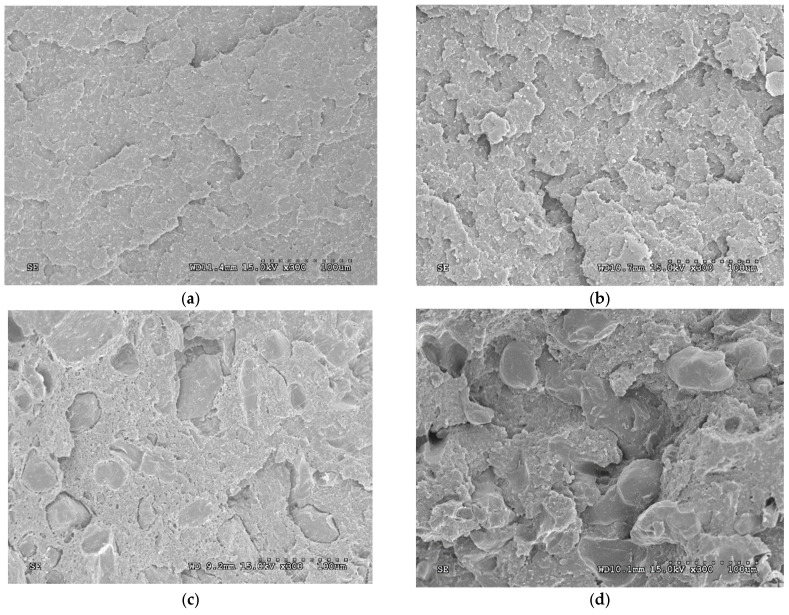
SEM micrographs of the impact fracture surfaces of the composites: (**a**) TPS after the first injection cycle; (**b**) TPS after the fourth injection cycle; (**c**) TPS/ASP biocomposite after the first injection cycle; (**d**) TPS/ASP biocomposite after the fourth injection cycle.

The fracture of the surface after impact was studied by SEM analysis. TPS surface after the first injection cycle (Figure 8a) showed less roughness than after the fourth injection cycle (TPS/ASP-4) (Figure 8b). This increase in the surface roughness can be directly attributed to the degradation of the TPS matrix. SEM micrographs of the impact fracture surfaces of TPS/ASP biocomposite gave qualitative information about the dispersion of almond shell particles. The fracture surfaces of TPS/ASP after the first injection cycle (Figure 8c) showed that the almond shell was homogeneously distributed in the polymer matrix. As with the TPS surface, it could be observed that the surface roughness and the imperfections on the impact fracture surface increased after the fourth injection cycle (TPS/ASP-4) (Figure 8d).

### 3.4. Melt Flow Index

**Figure 9:**
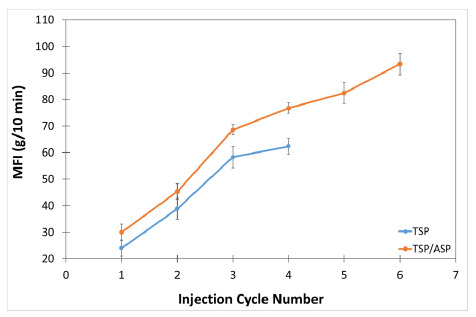
Variation of the melt flow index (MFI) after different reprocessing cycles.

It is a well-known fact that polymer chain breaking due to polymer degradation results in reduced molecular weight and increased flowability with temperature [20]. MFI is a simple test that can potentially offer detailed information about the degradation a polymer material has undergone [5,20]. Figure 9 shows the evolution of the MFI values after different reprocessing cycles. As can be seen, the MFI suffered a drastic increment in the value in both TPS and TPS/ASP biocomposite from the second injection process (TPS-2 and TPS/ASP-2). This effect was observed in other grades of commercial Mater-Bi and Mater-Bi YI014U/C, in which MFI increased from 9 to 16 g/10 min after the second processing [7]. TPS/ASP-1 biocomposite presented an MFI value higher than TPS-1, probably because TPS/ASP was previously subjected to a thermal process to obtain the pellets. However, despite the evident degradation of the material according to the MFI values obtained, this was not reflected in the decrease in the mechanical and thermal properties of the material.

## 4. Conclusions

This work describes the influence of the recycling, by injection molding reprocessing, of TPS and TPS/ASP biocomposite samples. To do so, the visual aspect, mechanical and thermal properties, and melt flow index were studied. TPS was processed four times and TPS/ASP biocomposite six times.

Experimental results revealed that reprocessing cycles had more effect on the TPS/ASP biocomposite. The change in color of TPS was 2.9 after all four cycles, meaning that only an experienced observer could notice the difference in color. However, after only two injection cycles, the change in color of TPS/ASP biocomposite was higher than unfilled TPS, and an observer could notice the change in color ($∆E_{ab}^{*}$ > 5).

Regarding the thermal properties, TGA and DSC analyses showed that after four injection cycles of TPS (TPS-4), the values remained almost invariable compared to TPS-1. This indicated that the reprocessing cycles did not have a significant effect on thermal degradation and main thermal transitions. On the other hand, in the case of TPS/ASP, in the second injection cycle, a significant reduction in T_onset_ (9 °C) was produced and to a lesser extent in the melting temperature (T_m1_ and T_m2_ decreased in 2–3 °C respect to TPS/ASP-1). The values continued to decrease progressively after each reprocessing cycle.

Moreover, it was observed that the reprocessing cycles had an influence on the mechanical properties of the TPS after the third processing cycle. In particular, Young’s modulus was reduced, indicating a loss of rigidity. Furthermore, after the fourth processing (TPS-4), the material lost ductility. In regard to the TPS/ASP biocomposite, the reprocessing did not have significant effects on tensile properties. The results maintained similar values to TPS/ASP-1 until the sixth cycle. As for impact strength, a slight reduction in energy absorption capacity was obtained after the second processing cycle (TPS-2), and no significant variations on the impact strength were observed with more cycles. However, TPS/ASP biocomposite showed an evident reduction in energy absorption capacity after the second processing cycle and continued when increasing reprocessing cycles. This confirmed the negative effect of biocomposite reprocessing on toughness.

Finally, the MFI showed a noticeable increase in both TPS and TPS/ASP biocomposites. Despite this, it was not reflected in such a drastic decrease in the mechanical and thermal properties of the material.

As a general conclusion, this study revealed that the TPS/ASP biocomposite is more sensitive than TPS to recycling regarding mechanical and thermal properties and visual aspects. From an industrial point of view, TPS could be reprocessed at least four times, as shown in this work, without the need of adding a virgin material. The TPS/ASP biocomposite could be recycled up to two or three cycles, but the impact strength has to be taken into account if it is a critical property for the product considered as it is the most affected property.

## Figures and Tables

**Table 1:** Properties of Mater-Bi^®^ EI51N0 extract to datasheet supplied by Novamont.

Property	Test	Value
Melting temperature (°C)	ASTM-D3418	167
Tensile strength at break (MPa)	ASTM-D638	39
Elongation at break (%)	ASTM-D638	2.5
Young Modulus (MPa)	ASTM-D638	2200

**Table 2:** Content of moisture, volatile matter, fixed carbon ash, and composition of the used almond shell powder (ASP).

	Moisture (%)	Volatile Matter (%)	Fixed Carbon (%)	Ash (%)	Hemicellulose (%)	Cellulose (%)	Lignin (%)
ASP	7.4	64.5	19.4	8.7	37	27	36

**Table 3:** Properties of epoxidized linseed oil (ELO) datasheet supplied by Traquisa.

Property	Value
Density at 20 °C (kg/m^3^)	1050–1060
Viscosity at 25 °C (cP)	8–12
Acid value (mg KOH/g)	≤1
Iodine content	≤5
Oxygen epoxide (%)	≥8

**Table 4:** Injection condition of starch-based polymer and biocomposite.

Parameters	TPS	TPS/ASP
Barrel profile (°C)	210-190-170-140-50	200-190-170-140-50
Mold temperature (°C)	35	35
Injection speed (rpm)	60	60
Pack pressure (bar)	400	400
Pack time (s)	15	15
Back pressure (bar)	50	50
Cooling time (s)	40	40

**Table 5:** Nomenclature of recycled biocomposite.

Injection Cycle Number	Recycling Order	Designation of As-Received Material	Designation of Biocomposites
1	0	TPS-1	TPS/ASP-1
2	1	TPS-2	TPS/ASP-2
3	2	TPS-3	TPS/ASP-3
4	3	TPS-4	TPS/ASP-4
5	4	---	TPS/ASP-5
6	5	---	TPS/ASP-6

**Table 6:** Color parameters ($L^{*},\;a^{*},\;b^{*}$ and ${\Delta E}_{ab}^{*}$ injection-molded samples of starch thermoplastic polymer (TPS) and TPS/ASP biocomposite subjected to different reprocessing cycles.

Samples	$L^{*}$	$a^{*}$	$b^{*}$	$\Delta E_{ab}^{*}$
TPS-1	92.50 ± 0.48	0.72 ± 0.19	4.69 ± 0.16	---
TPS-2	91.76 ± 0.26	0.81 ± 0.05	4.69 ± 0.16	1.00
TPS-3	90.85 ± 0.16	0.65 ± 0.05	4.98 ± 0.16	1.91
TPS-4	89.88 ± 0.27	0.51 ± 0.03	5.28 ± 0.16	2.91
TPS/ASP-1	57.35 ± 0.74	6.61 ± 0.40	10.77 ± 0.18	---
TPS/ASP-2	53.95 ± 0.41	5.79 ± 0.10	8.92 ± 0.45	3.96
TPS/ASP-3	52.70 ± 0.44	5.96 ± 0.06	9.18 ± 0.19	4.96
TPS/ASP-4	50.25 ± 0.21	5.92 ± 0.03	9.25 ± 0.21	7.29
TPS/ASP-5	49.52 ± 0.63	5.82 ± 0.08	9.56 ± 0.37	7.96
TPS/ASP-6	47.90 ± 0.72	5.98 ± 0.15	9.92 ± 0.18	9.51

**Table 7:** Thermal properties of the injection-molded samples of TPS and TPS/ASP biocomposite subjected to different reprocessing cycles obtained by thermogravimetric analysis (TGA).

Samples	T_onset_ (°C)	T_Max1_ (°C)	T_Max2_ (°C)	Residual Weight (%)
TPS-1	326.1 ± 0.4	362.1 ± 0.3	405.6 ± 1.7	1.42 ± 0.04
TPS-2	327.9 ± 0.1	360.9 ± 0.2	409.6 ± 2.7	1.45 ± 0.02
TPS-3	325.9 ± 0.3	360.7 ± 1.2	409.3 ± 2.0	1.66 ± 0.30
TPS-4	325.3 ± 1.6	360.2 ± 1.4	409.5 ± 0.6	1.42 ± 0.03
TPS/ASP-1	315.0 ± 3.4	346.0 ± 0.5	406.9 ± 1.0	1.28 ± 0.04
TPS/ASP-2	305.9 ± 1.2	345.4 ± 1.4	407.6 ± 1.0	1.37 ± 0.06
TPS/ASP-3	303.9 ± 0.9	345.4 ± 0.5	406.9 ± 1.0	1.23 ± 0.01
TPS/ASP-4	299.4 ± 0.3	345.0 ± 0.9	406.5 ± 0.5	1.31 ± 0.06
TPS/ASP-5	300.4 ± 1.1	340.6 ± 2.2	407.6 ± 0.6	1.26 ± 0.22
TPS/ASP-6	298.8 ± 0.9	333.9 ± 0.2	405.7 ± 1.6	1.24 ± 0.09

**Table 8:** Thermal properties of the injection-molded samples of TPS and TPS/ASP biocomposite subjected to different reprocessing cycles obtained by differential scanning calorimetry (DSC).

Samples	T_c_ (°C)	∆H_c_ (J/g^−1^)	T_m1_ (°C)	T_m2_ (°C)	∆H_f_ (J/g^−1^)
TPS-1	109.5 ± 0.2	22.6 ± 1.1	161.4 ± 0.4	168.5 ± 0.1	23.8 ± 1.6
TPS-2	110.3 ± 0.3	23.4 ± 0.7	161.3 ± 0.2	168.4 ± 0.1	24.9 ± 0.6
TPS-3	110.4 ± 0.1	23.1 ± 0.7	161.1 ± 0.1	168.2 ± 0.1	25.2 ± 0.6
TPS-4	110.4 ± 0.3	23.3 ± 0.3	160.8 ± 0.2	167.6 ± 0.1	24.0 ± 1.0
TPS/ASP-1	104.6 ± 0.2	16.0 ± 1.2	157.1 ± 0.4	166.4 ± 0.4	17.2 ± 1.4
TPS/ASP-2	102.3 ± 0.8	15.9 ± 0.1	153.9 ± 0.5	164.6 ± 0.4	18.0 ± 0.2
TPS/ASP-3	100.5 ± 0.5	15.1 ± 0.2	152.7 ± 0.5	164.0 ± 0.1	17.6 ± 0.8
TPS/ASP-4	98.6 ± 1.7	14.2 ± 1.7	150.8 ± 0.4	163.1 ± 0.8	18.2 ± 0.4
TPS/ASP-5	98.4 ± 0.2	14.9 ± 0.9	150.8 ± 0.3	162.6 ± 0.1	18.1 ± 0.3
TPS/ASP-6	94.0 ± 0.4	13.5 ± 1.5	144.6 ± 0.3	162.7 ± 0.7	17.1 ± 1.3

**Table 9:** Mechanical properties, Young’s modulus (E), tensile strength (σ_M_), elongation at tensile strength (Ɛ_M_), tensile strength at break (σ_R_), elongation at break (Ɛ_R_), of TPS and TPS/ASP biocomposite subjected to different reprocessing cycles.

Samples	Young’s Modulus (MPa)	σ_M_ (MPa)	Ɛ_M_ (%)	σ_R_ (MPa)	Ɛ_R_ (%)
TPS-1	1658 ± 52	39.4 ± 0.7	2.8 ± 0.1	17.5 ± 0.7	13.0 ± 1.6
TPS-2	1570 ± 78	36.5 ± 0.5	2.7 ± 0.1	16.9 ± 2.7	10.7 ± 1.2
TPS-3	1184 ± 79	36.6 ± 0.7	3.1 ± 0.1	17.2 ± 0.5	10.7 ± 1.2
TPS-4	1108 ± 58	34.2 ± 0.5	3.1 ± 0.1	33.4 ± 1.5	3.3 ± 0.1
TPS/ASP-1	1050 ± 33	16.8 ± 0.2	2.2 ± 0.1	11.7 ± 0.4	7.7 ± 2.3
TPS/ASP-2	1040 ± 40	16.6 ± 0.4	2.1 ± 0.1	11.2 ± 0.5	6.8 ± 0.7
TPS/ASP-3	1057 ± 83	16.8 ± 0.2	2.1 ± 0.1	13.2 ± 0.2	4.3 ± 0.5
TPS/ASP-4	1066 ± 17	16.7 ± 0.1	2.3 ± 0.1	16.7 ± 0.1	2.3 ± 0.1
TPS/ASP-5	1028 ± 41	15.1 ± 0.4	1.8 ± 0.1	15.1 ± 0.4	1.8 ± 0.1
TPS/ASP-6	1052 ± 47	13.4 ± 0.9	1.5 ± 0.1	13.4 ± 0.9	1.5 ± 0.1

## Data Availability

The data presented in this study are available on request from the author.
